# Late-Onset Rapidly Progressive Spastic Paraplegia with Extensive White Matter Abnormalities Associated with an MFN2 Variant

**DOI:** 10.3390/neurosci7040082

**Published:** 2026-07-17

**Authors:** Jiwon Yang, Hyeon-Mi Park, Yeong-Bae Lee

**Affiliations:** Department of Neurology, Gil Medical Center, Gachon University College of Medicine, Incheon 21565, Republic of Korea; jiwonyang@gachon.ac.kr (J.Y.); neurohm@gilhospital.com (H.-M.P.)

**Keywords:** Charcot–Marie–Tooth disease, axonal, type 2A1, spastic paraplegia, mitochondria

## Abstract

Mitofusin-2 (MFN2) variants are a well-established cause of Charcot–Marie–Tooth disease type 2A, although central nervous system involvement has increasingly been recognized in a subset of affected patients. We report a 51-year-old woman carrying a likely pathogenic MFN2 variant (c.2119C>T, p.Arg707Trp) who developed rapidly progressive spastic paraplegia and became wheelchair-dependent within several months. Neurological examination demonstrated severe pyramidal tract signs with preserved sensory function. Nerve conduction studies were suggestive of distal motor axonal involvement, while transcranial magnetic stimulation and somatosensory evoked potentials indicated corticospinal and central sensory pathway dysfunction in the lower extremities. Brain magnetic resonance imaging revealed extensive bilateral confluent periventricular and deep white matter hyperintensities. Comprehensive investigations excluded inflammatory, vascular, metabolic, infectious, neoplastic, and common genetic causes of hereditary spastic paraplegia. Targeted next-generation sequencing identified a heterozygous likely pathogenic MFN2 p.Arg707Trp variant. Although central nervous system manifestations have previously been described in MFN2-related disease, this phenotype is unusual because of the combination of late-onset rapidly progressive spastic paraplegia, and extensive cerebral white matter abnormalities associated with the p.Arg707Trp variant. This case further expands the recognized phenotypic spectrum of MFN2-related disease and highlights that MFN2 variants should be considered in the differential diagnosis of selected patients with late-onset progressive spastic paraplegia accompanied by cerebral white matter abnormalities and distal motor axonal neuropathy.

## 1. Introduction and Clinical Significance

Mitofusin-2 (MFN2) is a mitochondrial outer membrane protein involved in mitochondrial fusion. Pathogenic variants in MFN2 are a well-established cause of axonal Charcot–Marie–Tooth disease type 2A (CMT2A), which typically presents with progressive distal motor and sensory neuropathy with relatively preserved nerve conduction velocities [1]. However, clinical variability has been recognized, including cases with pyramidal tract signs (historically classified as hereditary motor and sensory neuropathy type V [HMSN V]) and optic atrophy (HMSN VI), suggesting that MFN2-related disorders may demonstrate substantial phenotypic heterogeneity beyond classical peripheral neuropathy [2].

Here, we report a patient harboring a likely pathogenic MFN2 variant who presented with rapidly progressive spastic paraplegia, unusually extensive cerebral white matter abnormalities, and distal motor neuropathy.

## 2. Case Presentation

A 51-year-old woman presented with rapidly progressive gait impairment. Despite remaining ambulatory until 2024 with only occasional falls, she developed progressive gait difficulty in January 2025 and became wheelchair-dependent within several months. She had no history of hypertension or diabetes mellitus; her medical history was notable only for hypothyroidism. She reported drinking alcohol three times per week over the past three years, with no prior history of regular alcohol use. There was no family history of neuropathy, gait disturbance, early wheelchair dependence, visual impairment, or other known neurological disorders. She denied visual symptoms. Physical examination revealed no clinical apparent lipomatosis or lipodystrophy.

On neurological examination, she was alert and able to cooperate, with no evidence of delirium or psychiatric symptoms. Cognitive screening revealed a Mini-Mental State Examination score of 17/30. Cranial nerve examination was otherwise unremarkable. Motor strength was preserved in the upper extremities, whereas the lower extremities showed paraplegia with severe lower-extremity contractures. Only trace voluntary movement was observed at the ankles and toes; movements at the hips and knees were restricted by contractures, precluding reliable quantification of lower-limb spasticity using the Modified Ashworth Scale. Retrospective assessment using the Spastic Paraplegia Rating Scale demonstrated complete loss of gait, stair climbing, and chair-rising abilities (4 points each), together with severe lower-limb contractures (4 points). Deep tendon reflexes were diffusely hyperactive in all extremities. Hoffmann signs were equivocal bilaterally. Bilateral extensor plantar responses and sustained ankle clonus were present. Sensory examination was unremarkable.

**Figure 1:**
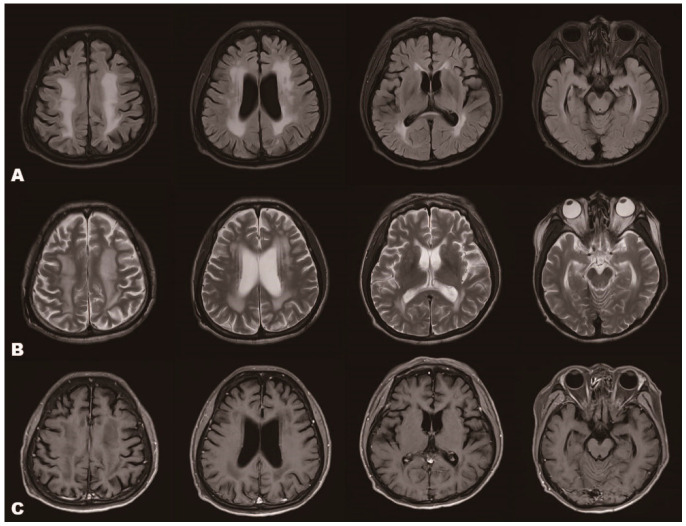
Brain magnetic resonance imaging of the patient. (**A**) Axial fluid-attenuated inversion recovery (FLAIR) images. (**B**) Axial T2-weighted images. (**C**) Axial T1-weighted images.

Nerve conduction studies (NCSs) revealed normal amplitudes of compound muscle action potential (CMAP) and sensory nerve action potential (SNAP) and normal conduction velocities (CVs) in the bilateral median nerve, ulnar nerve, and sural nerve. In the posterior tibial nerves, motor CVs were 41.6 m/s (right) and 43.1 m/s (left), with CMAP amplitudes of 5.0 mV and 6.3 mV, respectively, both of which were at the lower end of the laboratory reference range. The peroneal nerves showed motor CVs of 40.1 m/s (right) and 39.7 m/s (left), with markedly reduced CMAP amplitudes of 1.6 mV and 0.5 mV, respectively, suggesting predominant distal motor axonal involvement. Needle electromyography of the lower extremities was partially performed. No abnormal spontaneous activity was observed in the muscles examined; however, reliable evaluation of motor unit action potential and recruitment pattern was not feasible because severe lower-extremity weakness and contractures precluded sufficient voluntary muscle activation. Transcranial magnetic stimulation failed to elicit motor evoked potentials (MEPs) in the abductor hallucis brevis, suggesting impaired corticospinal conduction to the lower limbs. Posterior tibial somatosensory evoked potentials (SEPs) demonstrated preserved lumbar (L3/T12) responses with poorly formed cortical P37 waveforms, suggesting central sensory pathway involvement, whereas median nerve SEPs were normal (Supplementary Figures S1–S3). Brain magnetic resonance imaging (MRI) demonstrated bilateral symmetric confluent T2- and fluid-attenuation inversion recovery hyperintensities predominantly involving the periventricular and deep fronto-parietal white matter, without restricted diffusion, gadolinium enhancement, cortical involvement, mass effect, or vascular territorial distribution. Mild enlargement of the lateral ventricles appeared disproportionate to the patient’s age (Figure 1). Extensive evaluation for inflammatory, vascular, metabolic, infectious, and neoplastic etiologies, including CSF analysis, autoimmune/paraneoplastic antibody testing, vitamin and trace element studies, and spine MRI, was unrevealing. Targeted next-generation sequencing (NGS) was performed using a custom gene panel targeting 4873 genes on the Illumina NextSeq 550Dx platform (Illumina, San Diego, CA, USA), and variants were interpreted according to the American College of Medical Genetics and Genomics/Association for Molecular Pathology (ACMG/AMP) guidelines. No pathogenic variants were identified in genes associated with hereditary spastic paraplegia (HSP) or NOTCH3-related arteriopathy. However, a heterozygous MFN2 variant (c.2119C>T, p.Arg707Trp), which was classified as likely pathogenic according to the ACMG/AMP guidelines. According to the clinical laboratory’s standard workflow, pathogenic and likely pathogenic variants identified by NGS were confirmed by Sanger sequencing. The variant had a CADD PHRED score of 29.3 and a REVEL score of 0.84, providing additional computational evidence supporting its pathogenicity. This variant, located within the heptad repeat 2 (HR2) domain of MFN2, has been reported in association with Charcot–Marie–Tooth disease type 2A, a predominantly autosomal dominant axonal neuropathy [3]. No additional pathogenic variants were detected. Although the family history was unremarkable, genetic testing of family members was not performed; therefore segregation analysis could not be undertaken.

Brain magnetic resonance imaging revealed symmetric confluent hyperintense signal changes in the bilateral periventricular and deep white matter on FLAIR and T2-weighted images. The lesions predominantly involved the frontoparietal white matter. No focal mass effect was observed. T1-weighted images showed corresponding hypointense signal changes. No contrast enhancement was observed on post-contrast T1-weighted images.

## 3. Discussion

The present case was characterized by several notable clinical features, including rapid progression to wheelchair dependence, relatively late onset despite severe disability, upper motor neuron-predominant findings with electrophysiological findings suggestive of distal motor axonal involvement and sensory nerve sparing, and extensive confluent white matter abnormalities, in the context of a likely pathogenic MFN2 gene variant. Although central nervous system (CNS) involvement has previously been reported in MFN2-related disease, the phenotype observed in our patient extends the known clinical spectrum. To our knowledge, this combination of rapidly progressive spastic paraplegia, and extensive confluent cerebral white matter abnormalities has not previously been reported in association with the MFN2 p.Arg707Trp variant.

MFN2-related disease shows marked phenotypic variability, including considerable intrafamilial and interfamilial variability, with variable age at onset and disease severity ranging from mild to severe disabling peripheral neuropathy [2,3,4,5,6,7,8]. Additional manifestations include optic atrophy, spastic paraparesis, cognitive dysfunction, hearing loss, and brain MRI abnormalities, suggesting that MFN2-related disease may involve both peripheral and central nervous systems [2,3,9,10]. Although CNS involvement has been increasingly recognized, its frequency and severity vary substantially across published cohorts. To better contextualize our findings, previously reported MFN2-related CNS phenotypes are summarized in Table 1, while the reported clinical spectrum associated specifically with the MFN2 p.Arg707Trp variant is summarized in Supplementary Table S1.

Late-onset spastic paraplegia encompasses a broad differential diagnosis, including common genetic forms of HSP particularly SPG4, the most common autosomal dominant form of HSP, as well as SPG7, SPG11, and SPG15, and acquired disorders including primary progressive multiple sclerosis, cervical spondylotic myelopathy, adrenomyeloneuropathy, and metabolic or inflammatory myelopathies. In the present patient, extensive evaluation excluded structural, inflammatory, vascular, metabolic, and common genetic causes of HSP, whereas targeted neuropathy gene panel testing identified the likely pathogenic MFN2 p.Arg707Trp variant. This case highlights that MFN2-related disease should be considered in the differential diagnosis of late-onset progressive spastic paraplegia, particularly when accompanied by distal motor axonal neuropathy or cerebral white matter abnormalities. MFN2 encodes a mitochondrial outer membrane GTPase and contributes to mitochondrial network organization through mitochondrial fusion. Mitochondria are transported along axons via microtubule-based motor proteins such as kinesin and dynein and accumulate at the sites of high energy demand, such as the axon initial segment, nodes of Ranvier, and synaptic terminals, including the neuromuscular junction [1,14]. Disruption of this process has been implicated in mitochondrial transport defects in long neuronal projections. The identified variant (p.Arg707Trp) is located within the heptad repeat 2 (HR2) domain of MFN2, a region critical for mitochondrial tethering and fusion through coiled –coil interactions between adjacent mitochondria [15,16]. Variants affecting this region may disrupt mitochondrial dynamics and impair axonal transport, providing a plausible biological basis for the combined central and peripheral motor involvement observed in our patient [16].

Although mitochondrial trafficking defects associated with MFN2 dysfunction may increase the vulnerability of neurons with long axonal projections, this mechanism primarily explains length-dependent peripheral axonal neuropathy and does not fully account for the white matter abnormalities observed on brain MRI. The pathophysiological basis of cerebral white matter changes in MFN2-related disease remains unclear. These abnormalities may reflect metabolic vulnerability of glial cells or reactive gliosis associated with mitochondrial dysfunction. Oligodendrocytes are increasingly recognized as critical regulators of axonal energy homeostasis through metabolic support of myelinated axons, and impaired axon–glial metabolic coupling may contribute to white matter dysfunction even in the absence of overt demyelination [17]. Prior proton MRS studies in MFN2-related CMT2 with cerebral involvement suggested reactive gliosis with little evidence of cerebral axonal loss and only mild pyramidal signs [9]. However, experimental MFN2 R94Q models demonstrate diffuse axon degeneration in central white matter tracts, including pyramidal pathways, accompanied by reactive astro- and microgliosis without neuronal cell body loss. Experimental models of hereditary spastic paraplegia have demonstrated accumulation of dysfunctional axonal mitochondria despite preserved myelin integrity, supporting a potential role of mitochondrial-mediated axoglial dysfunction in central motor tract degeneration [18,19]. Together, these findings suggest that CNS involvement in MFN2-related disease may occur with variable pathological substrates and clinical severity. Although the electrophysiological abnormalities were relatively mild compared with the severity of the patient’s spastic paraplegia, borderline tibial CMAP amplitudes, together with the markedly reduced peroneal CMAP amplitudes, were suggestive of distal motor axonal involvement in the lower extremities.

This study has several limitations. First, segregation analysis could not be performed because genetic testing was unavailable for the patient’s family members. Although no family history suggestive of hereditary neurological disease was identified, the pathogenicity of the identified MFN2 variant could not be further supported by co-segregation analysis. Second, although the patient did not report visual symptoms suggestive of optic nerve involvement, ophthalmologic assessments, including optical coherence tomography and visual evoked potentials, were not performed. Therefore, subclinical optic pathway involvement cannot be completely excluded. Third, this is a single case report, precluding definitive conclusions regarding genotype–phenotype correlations or the mechanism underlying CNS involvement. Further studies involving additional patients and functional analyses are needed to better define the phenotypic spectrum associated with the MFN2 p.Arg707Trp variant.

## 4. Conclusions

Taken together, this case further expands the recognized clinical spectrum of MFN2-related disorders and highlights the MFN2 p.Arg707Trp variant by demonstrating an unusual combination of rapidly progressive spastic paraplegia, severe corticospinal tract dysfunction, and extensive cerebral white matter abnormalities. Our findings suggest that MFN2-related disease may be considered in the differential diagnosis of selected patients with late-onset progressive spastic paraplegia accompanied by combined central and peripheral motor system involvement.

## Figures and Tables

**Table 1:** Reported central nervous system manifestations associated with MFN2 mutations and comparison with the present case.

Reference	MFN2 Variant (cDNA, Protein Change)	Patients with CNS Involvement (n/N) *	Age at Onset	CNS Involvement	Brain MRI Findings
Chung et al. (2006) [6]	c.1090C>T (p.Arg364Trp); c.839G>A (p.Arg280His); c.1085C>T (p.Thr362Met); c.494A>G (p.His165Arg); c.380G>A (p.Gly127Asp)	8/21	One early-onset; seven late-onset	Transient dysarthria, migraine, transient hemisensory symptoms, bilateral extensor plantar responses, sensorineural hearing loss, or isolated MRI abnormalities	Periventricular and subcortical white matter hyperintensities
Züchner et al. (2006) [11]	c.280C>T (p.Arg94Trp), c.617C>T (p.Thr206Ile), c.827A>G (p.Gln276Arg), c.1081C>T (p.His361Tyr), c.1090C>T (p.Arg364Trp), c.1252C>T (p.Arg418Ter)	10/10	Neuropathy: 1–10 years; optic atrophy: 5–50 years	Bilateral optic atrophy with severe visual impairment	Bilateral T2 hyperintensity of the cerebellar peduncles (1/10)
Brockmann et al. (2008) [7]	c.310C>T (p.Arg104Trp); IVS5-1G>C (splice-site); c.1132T>C (p.Ser378Pro)	4/4	Early onset (3–7 years)	Optic atrophy (2/4), secondary macrocephaly (2/4), exaggerated deep tendon reflexes/pyramidal signs (2/4), saccadic interruption of horizontal eye movements (1/4)	Mild diffuse periventricular parieto-occipital white matter hyperintensities (2/4), bilateral thalamic T2 hyperintensities (1/4)
Del Bo et al. (2008) [2]	c.310C>T (p.Arg104Trp)	3/3	Early onset (2–14 years)	Cognitive impairment, pyramidal signs/spastic paraparesis, optic nerve dysfunction/optic neuropathy, sensorineural hearing loss	Focal telencephalic white matter lesions in the adult proband (1/3)
Chung et al. (2010) [4]	c.280C>T (p.Arg94Trp), c.494A>G (p.His165Arg), c.1048T>C (p.Ser350Pro), c.1090C>T (p.Arg364Trp)	7/18	Early-onset (<10 years)	Optic atrophy, vocal cord paralysis, transient sensory symptoms; one patient developed brain lesions before peripheral neuropathy	Multiple periventricular/subcortical white matter hyperintensities; occasional brainstem, cerebellar, and dentate nucleus involvement.
Klein et al. (2011) [8]	c.436C>T (p.Leu146Phe)	2/10	1–45 years (marked intrafamilial variability)	Optic atrophy, one patient was initially misdiagnosed with multiple sclerosis	Paraventricular white matter T2 hyperintensities (1/10)
Rouzier et al. (2012) [12]	c.629A>T (p.Asp210Val)	2/11	Optic atrophy: early childhood; neuropathy: 10–54 years	Optic atrophy, pyramidal syndrome/cerebellar ataxia (adult), spastic paraparesis and psychomotor regression (child)	Periventricular white matter lesions with diffuse cerebral atrophy (adult); periventricular/subcortical leukodystrophy (child).
Hayashi et al. (2023) [13]	c.1090C>T (p.Arg364Trp)	2/2	Early childhood (2–5 years)	Optic pathway involvement, spastic paraparesis, pyramidal tract involvement	Subcortical white matter hyperintensities, middle cerebellar peduncle/cerebellar white matter hyperintensities, and optic pathway atrophy.
Present case	c.2119C>T (p.Arg707Trp)	1/1	Adult-onset (51 years)	Rapidly progressive spastic paraparesis, pyramidal tract signs, mild axonal neuropathy	Extensive confluent bilateral white matter hyperintensities

* Values are presented as the number of patients with reported CNS manifestations/total number of patients, according to each original study. Only MFN2 variant(s) associated with the reported CNS manifestations are listed for each study.

## Data Availability

The data presented in this study are not publicly available due to patient privacy and ethical restrictions but are available from the corresponding author upon reasonable request.

## Supplementary Materials

The following supporting information can be downloaded at: https://www.mdpi.com/article/10.3390/neurosci7040082/s1, Figure S1: Motor evoked potentials (MEPs); Figure S2: Posterior tibial somatosensory evoked potentials (SEPs); Figure S3: Median nerve somatosensory evoked potentials (SEPs); Table S1: Clinical phenotypes associated with the MFN2 p.Arg707Trp (R707W) variant.

## Abbreviations

The following abbreviations are used in this manuscript:

MFN2	Mitofusin-2
CMT2A	Charcot–Marie–Tooth disease type 2A
HMSN V	Hereditary motor and sensory neuropathy type V
NCSs	Nerve conduction studies
CMAP	Compound muscle action potential
SNAP	Sensory nerve action potential
CVs	Conduction velocities
MEPs	Motor evoked potentials
SEPs	Somatosensory evoked potentials
NGS	Next-generation sequencing
HSP	Hereditary spastic paraplegia
ACMG/AMP	American College of Medical Genetics and Genomics/Association for Molecular Pathology
CADD	Combined Annotation Dependent Deletion
REVEL	Rare Exome Variant Ensemble Learner
HR2	Heptad-repeat
