# Supplementary material for: Late-Onset Rapidly Progressive Spastic Paraplegia with Extensive White Matter Abnormalities Associated with an MFN2 Variant
Source: NeuroSci. 2026 Jul 17;7(4):82. doi: 10.3390/neurosci7040082 (PMC13397902; doi:10.3390/neurosci7040082)
Supplement: Supplementary file 1 [file neurosci-07-00082-s001.zip › neurosci-4354895-supplementary.pdf]

Supplementary Figures

Magnetic stim(MagPro)

	Latency ms	Ampl. mV	Dur. ms	Area mVms
**R Tibial (Foot) - AH**				
100% STIMULI	**NR**	**NR**	**NR**	**NR**
**L Tibial (Foot) - AH**				
100% STIMULI	**NR**	**NR**	**NR**	**NR**

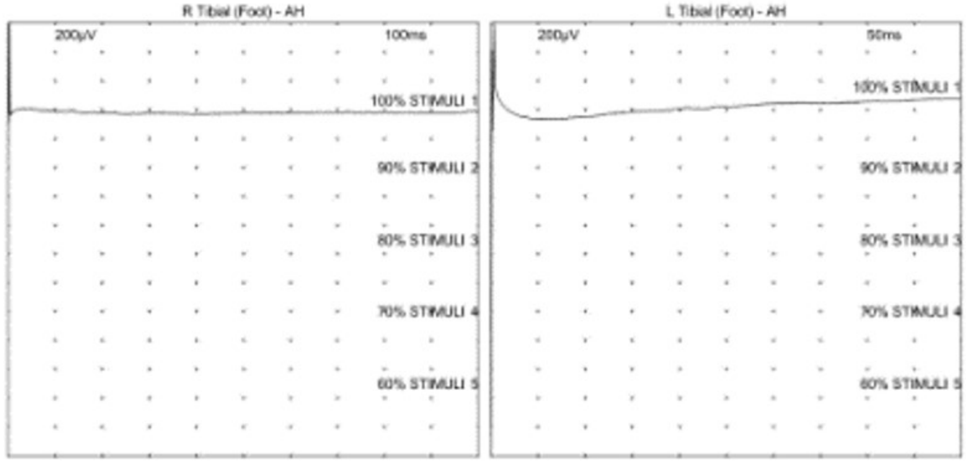

Figure S1. Motor evoked potentials (MEPs).

Motor evoked potentials recorded from the abductor hallucis (AH) muscles showed no reproducible responses bilaterally despite maximal stimulation.

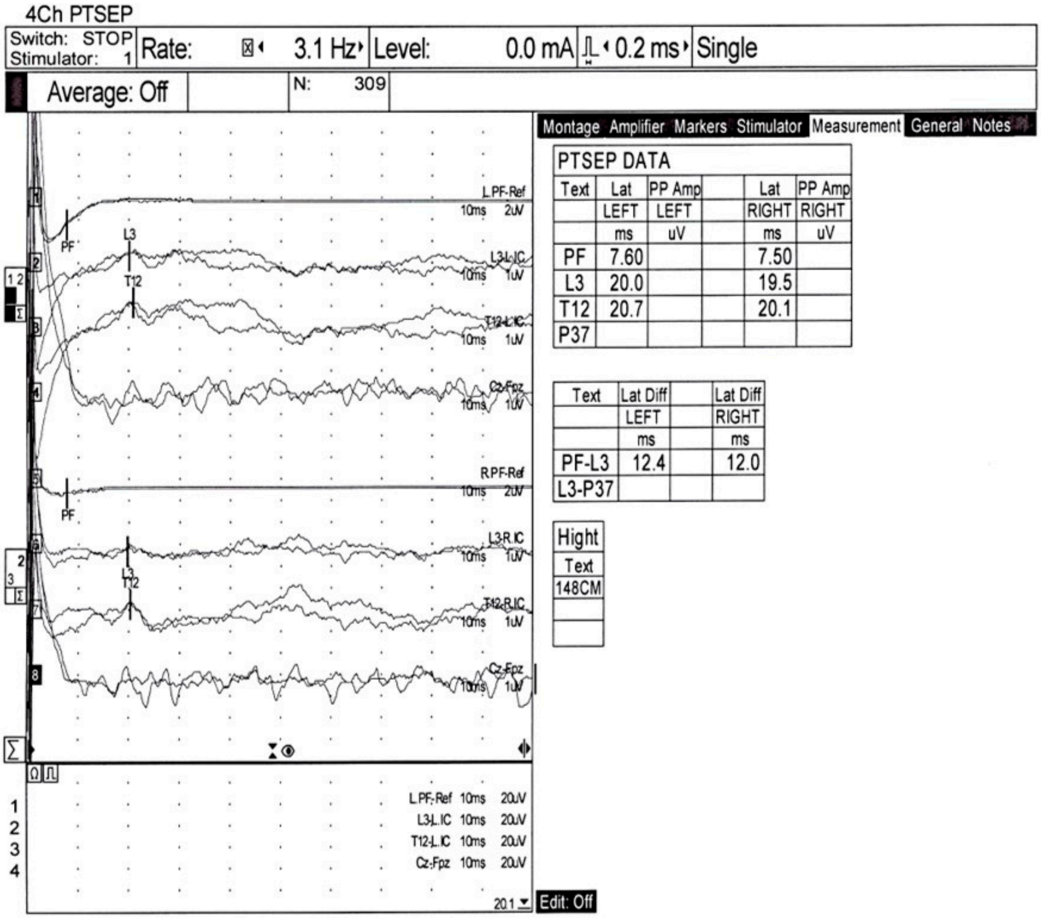

Figure S2. Posterior tibial somatosensory evoked potentials (SEPs).

Posterior tibial nerve SEPs showed preserved peripheral and spinal responses, with identifiable potentials at the popliteal fossa (PF) and lumbar (L3/T12) levels bilaterally. However, cortical responses (P37) were absent bilaterally

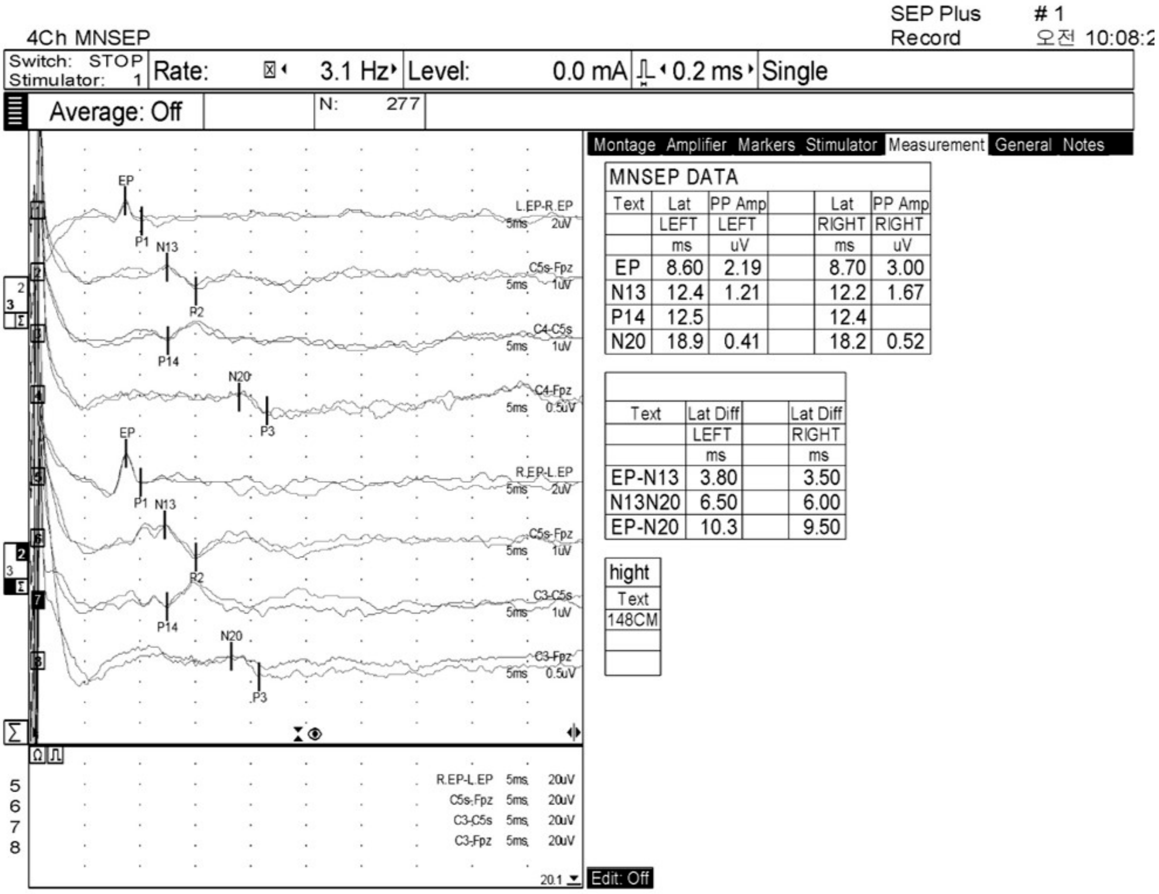

Figure S3. Median nerve somatosensory evoked potentials (SEPs).

Normal responses were recorded at Erb’s point (EP), cervical (N13), and cortical (N20) levels bilaterally, indicating preserved peripheral and central sensory conduction in the upper limbs.

Supplementary Table S1. Clinical phenotypes associated with the MFN2 p.Arg707Trp (R707W) variant

References	Allelic status	Inheritance	Major clinical features
Nicholson et al. (2008) [1]	Homozygous	AR	Severe early-onset axonal neuropathy with lipodystrophy
Calvo et al. (2009) [2]	Compound heterozygous	AR	Moderate childhood-onset CMT2
Braathen et al. (2010) [3]	Not explicitly stated*	AD	Late-onset CMT2 (onset 44 years)
Sawyer et al. (2015) [4]	Homozygous	AR	Adult-onset MSL with progressive axonal neuropathy
Rocha et al. (2017) [5]	Homozygous or compound heterozygous	AR	Childhood- to adult-onset MSL with or without axonal neuropathy
Bansagi et al. (2017) [6]	Heterozygous	Not reported	Late-onset distal hereditary motor neuropathy
Present case	Heterozygous	Sporadic	Rapidly progressive spastic paraplegia with extensive cerebral white matter abnormalities

* The pedigree is compatible with autosomal dominant inheritance, although the zygosity of the affected individual was not explicitly stated in the original publication.

MSL, multiple symmetric lipomatosis

References:

1. Nicholson, G.A.; Magdelaine, C.; Zhu, D.; Grew, S.; Ryan, M.M.; Sturtz, F.; Vallat, J.M.; Ouvrier, R.A. Severe early-onset axonal neuropathy with homozygous and compound heterozygous MFN2 mutations. Neurology 2008, 70, 1678–1681.
2. Calvo, J.; Funalot, B.; Ouvrier, R.A.; Lazaro, L.; Toutain, A.; De Mas, P.; Bouche, P.; Gilbert-Dussardier, B.; Arne-Bes, M.C.; Carriere, J.P.; et al. Genotype-phenotype correlations in Charcot-Marie-Tooth disease type 2 caused by mitofusin 2 mutations. Arch. Neurol. 2009, 66, 1511–1516.

3. Braathen, G.J.; Sand, J.C.; Lobato, A.; Hoyer, H.; Russell, M.B. MFN2 point mutations occur in 3.4% of Charcot-Marie-Tooth families. An investigation of 232 Norwegian CMT families. BMC Med. Genet. 2010, 11, 48.
4. Sawyer, S.L.; Ng, A.C.-H.; Innes, A.M.; Wagner, J.D.; Dyment, D.A.; Tetreault, M.; Care, C.; Majewski, J.; Boycott, K.M.; Screaton, R.A.; et al. Homozygous mutations in MFN2 cause multiple symmetric lipomatosis associated with neuropathy. Hum. Mol. Genet. 2015, 24, 5109–5114.
5. Rocha, N.; Bulger, D.A.; Frontini, A.; Titheradge, H.; Gribsholt, S.B.; Knox, R.; Page, M.; Harris, J.; Payne, F.; Adams, C.; et al. Human biallelic MFN2 mutations induce mitochondrial dysfunction, upper body adipose hyperplasia, and suppression of leptin expression. eLife 2017, 6, e23813.
6. Bansagi, B.; Griffin, H.; Whittaker, R.G.; Antoniadi, T.; Evangelista, T.; Miller, J.; Greenslade, M.; Forester, N.; Duff, J.; Bradshaw, A.; et al. Genetic heterogeneity of motor neuropathies. Neurology 2017, 88, 1226–1234.
